# An Improved New YOLOv7 Algorithm for Detecting Building Air Conditioner External Units from Street View Images

**DOI:** 10.3390/s23229118

**Published:** 2023-11-11

**Authors:** Zhongmin Tian, Fei Yang, Donghong Qin

**Affiliations:** 1 College of Artificial Intelligence, Guangxi Minzu University, Nanning 530006, China; 2 State Key Laboratory of Resources and Environmental Information System, Institute of Geographic Sciences and Natural Resources Research of Chinese Academy of Sciences, Beijing 100101, China; 3 Jiangsu Center for Collaborative Innovation in Geographical Information Resource Development and Application, Nanjing 210023, China

**Keywords:** YOLO, heat wave hazards, street view, air conditioner, detection algorithm

## Abstract

Street view images are emerging as new street-level sources of urban environmental information. Accurate detection and quantification of urban air conditioners is crucial for evaluating the resilience of urban residential areas to heat wave disasters and formulating effective disaster prevention policies. Utilizing street view image data to predict the spatial coverage of urban air conditioners offers a simple and effective solution. However, detecting and accurately counting air conditioners in complex street-view environments remains challenging. This study introduced 3D parameter-free attention and coordinate attention modules into the target detection process to enhance the extraction of detailed features of air conditioner external units. It also integrated a small target detection layer to address the challenge of detecting small target objects that are easily missed. As a result, an improved algorithm named SC4-YOLOv7 was developed for detecting and recognizing air conditioner external units in street view images. To validate this new algorithm, we extracted air conditioner external units from street view images of residential buildings in Guilin City, Guangxi Zhuang Autonomous Region, China. The results of the study demonstrated that SC4-YOLOv7 significantly improved the average accuracy of recognizing air conditioner external units in street view images from 87.93% to 91.21% compared to the original YOLOv7 method while maintaining a high speed of image recognition detection. The algorithm has the potential to be extended to various applications requiring small target detection, enabling reliable detection and recognition in real street environments.

## 1. Introduction

The frequency and intensity of heat wave disasters are increasing in the context of global warming, posing a severe threat to human health and socio-economic security [1,2,3]. Therefore, disaster prevention has become critical worldwide. Research has shown that air conditioning is an effective measure for coping with heat wave disasters [4,5]. The 2021 report of the Lancet Countdown on Health and Climate Change revealed that access to effective cooling through air conditioning had already saved tens of thousands of lives in 2019 [6,7].

Understanding the spatial distribution of air conditioner usage is crucial for accurately assessing residents’ capacity to cope with heat wave disasters and supporting local governments and policymakers in developing effective policies for disaster prevention and relief. However, traditional statistical methods for collecting air conditioning usage data, whether through direct surveys of residential areas or projections based on local air-conditioning statistics, are costly in terms of human resources, the accuracy of information collection is challenging to ensure, and obtaining spatial distribution data for air conditioning usage in the region is difficult. Thus, there is a critical need to develop a convenient, efficient, and simple method for extracting information on air conditioner usage and its spatial distribution.

In recent years, street view services like Google Street View, Baidu Panorama, and Tencent Street View have undergone gradual development and improvement. Consequently, the amount of street view image data has significantly increased, and the coverage has expanded to encompass more than 50% of the global population [8,9]. Street view images offer wide coverage and street-level landscape information, serving as a substantial data source and a catalyst for new research ideas in urban environmental evaluation studies. Street view images provide a real depiction of urban streets from a parallel human perspective, which is not attainable through remote sensing data sources like satellite and aerial images [10]. Hence, combining street view images with remote sensing images to extract indicator information represents an ideal approach [11].

Deep learning methods are rapidly advancing in the field of artificial intelligence, providing a means to extract classification features from data sets and achieve automatic and efficient target object classification [12]. Accurate recognition of targets in images is increasingly crucial [13]. In deep-learning-based target detection algorithms, mainstream approaches can be classified as one-stage detection algorithms (e.g., YOLO series) and two-stage detection algorithms (e.g., RCNN series), based on the number of stages in their algorithm structure. The two-stage detection algorithm consists of two target detection processes: candidate region generation and classification. While this approach improves accuracy, it also increases model complexity and time overhead, imposing limitations on computational efficiency [14]. On the other hand, single-stage algorithms directly apply algorithms to input images to generate both candidate regions and target classes through joint decoding. The YOLO algorithm [15], a classic and the first single-stage target detection algorithm, achieves satisfactory results in both detection accuracy and speed by adding classifiers and designing a new loss function to ensure the complete detection of multiple types of targets. Additionally, the YOLO algorithm incorporates advanced feature extraction network designs for efficient detection and lightweight overhead. Being the first deep-learning-based target detection algorithm to support real-time operation, the YOLO algorithm has garnered considerable attention.

The rapid advancements in deep learning methods have spurred a growing number of scholars to explore urban applications utilizing street view image data. Notably in urban planning, the application of deep learning methods to identify the high risk of soft-story buildings in street view images within seismic risk areas has significantly alleviated the challenges associated with traditional manual identification and statistical approaches. This approach enhances work efficiency and provides statistical results to support city managers and decision-makers in formulating strategies to mitigate building risks [16,17,18]. The fusion of multi-source and multi-modal data, including street view image data, is a widely employed strategy to compensate for the limitations of other data sources and leverage the unique advantages of street view images. This approach significantly enhances accuracy in applied research, particularly in land-use-type classification and building extraction [19,20,21]. In addition to these macro-level urban planning applications, utilizing street view image data to discern object details is a common strategy for responding to specific urban scenarios. For example, Gebru et al. [22] utilized Google Street View image data to identify the make, model, and year of motor vehicles encountered in a specific neighborhood, totaling 22 million cars (8% of all cars in the U.S.). This analysis accurately estimated income, race, education, and voting patterns at the postcode and precinct level. Krylov et al. [23] utilized Google Street View images to accurately detect the locations of utility poles and traffic lights, achieving a high success rate of over 90% with an accuracy range of 2 m. Chen et al. [24] utilized street view image data from Baidu Panorama to analyze green landscape statistics in various cities in South China. Their research revealed a positive correlation between the number of green landscapes and GDP, as well as other socioeconomic indicators. This highlights the importance of developing public green spaces as the city’s economy expands. Nguyen et al. [25] utilized Google Street View image data to extract diverse indicators related to street greenery, crosswalks, and building types. Through statistical analysis and in conjunction with local health records, they revealed that regions with abundant street and sidewalk greenery exhibited a lower prevalence of obesity and diabetes. Additionally, Hu et al. [26] investigated multiple pedestrian collisions and gathered various road condition variables, including the number of lanes and pavement condition, using street view image data. Their objective was to explore the correlation between collisions and road infrastructure characteristics.

The use of air conditioning, as one of the most important and effective measures to cope with high temperatures, is limited in its role in heat wave disaster response strategies by the difficulty of accurately expressing spatial distribution. Despite the valuable information support provided by street view images from web maps, research on extracting residential air conditioner external units and managing heat wave disasters based on these images is lacking [27]. There are the following difficulties in identifying targets of air conditioner external units in street view image data:

(1) The information extraction algorithms for air conditioner external units have low accuracy;
(2) The street view images exhibit uneven lighting conditions and complex backgrounds;
(3) The varying sizes of air conditioner external units are displayed in the street view image, hindering the observation of small targets from a distance;
(4) The targets are often obscured by objects such as trees and burglar windows, resulting in varying sizes of obscured areas, which severely hinder detection and recognition.

This study aims to propose targeted improvements to the YOLOv7 algorithm [28], which is part of the YOLO series algorithms. These improvements involve the introduction of attention mechanisms in the feature extraction layer and the addition of a small target detection layer [29,30]. This aims to solve the difficult problems of severe occlusion, complex background, low accuracy, and difficulty in identifying small targets in the application of identification of air conditioner external units. By developing an improved YOLOv7 algorithm, this study aims to enhance the accuracy of detecting air conditioner external units in vast street view images. These improvements aim to provide methodological and data support for rapidly and accurately acquiring the spatial distribution of air conditioner use. They also contribute to the development of more rational heat wave disaster response tactics and ultimately enhance the efficiency of such responses.

## 2. Materials and Methods

### 2.1. Study Area and Data

Currently, Baidu Maps has collected panoramic data for streets in 652 cities in China, covering a mileage of 2,295,000 km, and in most cities, users can access the street view of the roads in Baidu Maps. In the developer mode of Baidu Maps, developers can use the API interface after converting the coordinate system according to the coordinate point to obtain the target location of the 360° street panoramic image or other arbitrary direction of any angle range of the street image—usually, the distance from the setting of the road network coordinates every 25 m positioning, and thus developers can obtain a complete, coherent street image data.

**Figure 1:**
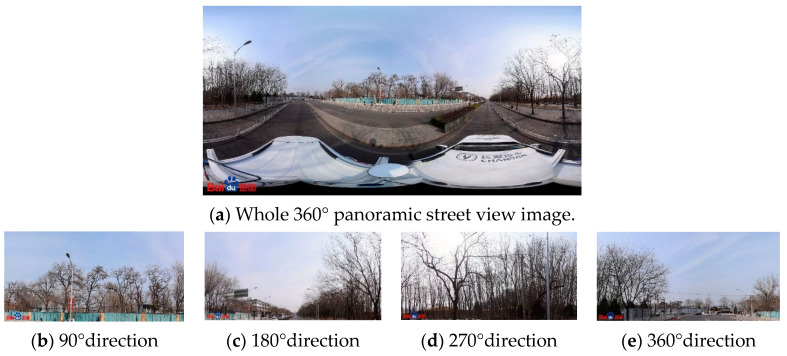
Example of frontal street view image orientation map.

The reproduction of a 360° panoramic image results in a degree of spatial distortion, which causes a distorted panoramic street view image (Figure 1a); as there is the choice of every 90° angle of four directions of the frontal street view image data, the image data obtained are more closely related to the real situation of the street scene (such as Figure 1b–d). Since the purpose of this study is to detect and count the air conditioner external units hanging on the walls of buildings, it is sufficient to choose only two directions of 90° and 270°, i.e., the left and right directions of the road.

This study focuses on the urban area of Guilin City, located in the Guangxi Zhuang Autonomous Region of China, as the study area for extracting air conditioner external units from street view image data on buildings along the roads. Guilin City, a renowned tourist destination, is situated in a subtropical region and experiences recurring high-temperature heat waves annually, posing a substantial threat to both local population and international visitors. As one of the initial cities chosen for China’s Sustainable Development Demonstration, Guilin is in immediate need of conducting research on high-impact urban disaster response and sustainable development. The construction of target detection models heavily relies on rich and rigorous data sets [31]. The street view images predominantly cover the main roads in Guilin City, with fixed points established every 25 m in the continuous urban main road network data, resulting in a total of 61,929 distinct road network coordinate points. By utilizing the acquired coordinate point information, a bulk collection of 123,858 street view images from both sides of the road was achieved through the Baidu panorama service. Subsequently, redundant street view images displaying excessive similarity were manually eliminated, resulting in the selection of 5680 instances of air conditioner external units as the training data set. The data set is comprehensive in its coverage, containing individual examples of air conditioner external units in a variety of states, including those obscured by trees and windows, those with small targets at a distance, and those placed in an oblique direction. In order to control the comprehensiveness and balance of the sample data set, we also manually screened the samples so that the number of samples of air-conditioning outboard units in each different state could occupy a large proportion. The screened street view images are annotated using the graphical image annotation tool ‘labelImg’ (https://github.com/tzutalin/labelImg, accessed on 8 October 2023). This tool identifies and frames all the air conditioner external units in each image, generating an XML file that contains the target type and coordinate information. The annotated data are then split in an 8:1:1 ratio for algorithm training, testing, and validation, respectively.

### 2.2. Data Pre-Processing and Data Augmentation

The 25 m distance interval set for the coordinates of the urban trunk road network ensures the continuity and integrity of the street view image data. However, in open areas, where there are few or no buildings nearby and highly overlapping scenes in the distance, it is necessary to pre-process the data by removing street view images that exhibit excessive similarity. This is completed to ensure data accuracy, minimize redundancy, and improve data utilization efficiency. Future research utilizing Street View image data will enable more accurate analysis and understanding of urban development.

**Figure 2:**
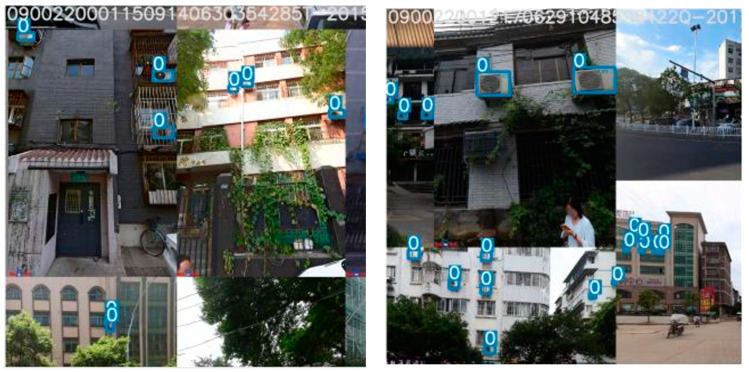
Mosaic data augmentation effect (“0” is the classification number of the air conditioner external unit).

Upon completing the data pre-processing, data augmentation is performed on the filtered data to mitigate potential overfitting issues. Data augmentation expands limited data to create more diverse training samples, thereby increasing both sample size and diversity, and improving model robustness. Given the characteristics of street view images, which may feature objects at varying distances, complex backgrounds, diverse lighting conditions, and occlusions, the Mosaic data augmentation method proposed in YOLOv4 [32] was chosen for this study. This approach combines four images into a single training sample, effectively reducing GPU memory usage and enhancing network robustness without requiring large mini-batch sizes. By randomly scaling and distributing images for stitching, Mosaic data augmentation enabled the synthesis of new image data with numerous small targets, and edge location labels assisted in identifying occluded objects, thereby enriching the detection data set. Figure 2 illustrates the results of this method.

### 2.3. Performance Evaluation Index

The performance evaluation indices consist primarily of mean average precision (MAP), precision (*P*), and recall (*R*). These metrics are calculated via the following formulas:

(1) $$P=\frac{T_{P}}{T_{P}+F_{P}}\times 100\%$$

(2) $$R=\frac{T_{P}}{T_{P}+F_{N}}\times 100\%$$

(3) $$AP=\int_{0}^{1}P\left(R\right)\;dR$$

where, *T_P_* represents the actual positive samples that were detected as positive, *F_P_* represents the actual negative samples that were erroneously detected as positive, and *F_N_* represents the actual negative samples that were correctly detected as negative. In turn, *P* corresponds to the ratio of correctly predicted outcomes to all predicted positive cases, whereas *R* pertains to the ratio of correctly predicted outcomes to all positive cases. The area under the *P*-*R* curve represents the *AP* value, with the mAP value calculated as the average *AP* values across all categories. To evaluate the detection performance of the complete target detection network model, the mAP value is commonly employed. For mAP@0.5, IoU (Intersection over Union) is established at 0.5, with the *AP* of all images in every category calculated and averaged across all categories. Similarly, mAP@0.5:0.95 is representative of the average mAP across distinct IoU thresholds (ranging from 0.5 to 0.95 in increments of 0.05).

The NMS method may not yield entirely accurate prediction frames and corresponding categories. Therefore, samples with confidence levels exceeding threshold a = 0.5 are identified as positive, and vice versa. Moreover, samples that overlap actual frames by an intersection ratio surpassing threshold *d* = 0.6 are designated as true positives (*T_P_*) while those that do not comprise false positives (*F_P_*). If any actual positive samples exist within the negative samples, they are categorized as false negatives (*F_N_*).

### 2.4. YOLO

This study selected three versions of the “You Only Look Once” (YOLO) series, namely YOLOv5, YOLOv7, and YOLOv8, as the primary algorithm choices. These single-stage target detection algorithms are preferred in current engineering applications due to the relative inefficiency of two-stage target detection algorithms like RCNN [33,34]. The experiments were conducted using a pre-processed data set of air conditioner external units. The detection results are presented in Table 1. It is evident from the results that YOLOv5 exhibits a slightly lower detection performance than the two latest versions, while the superiority of YOLOv8 over YOLOv7 is not significant. However, due to its recent release, YOLOv8 still lacks stability, and there are more unknown errors associated with implementing improvements and modifications compared to YOLOv7.

**Figure 3:**
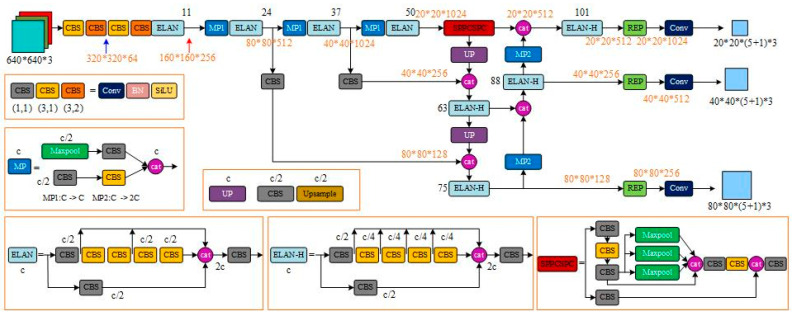
Original YOLOv7 network structure diagram.

Therefore, this study utilizes YOLOv7 as its foundation, which is the most reliable iteration of the YOLO series to date. The ELAN network [35], an efficient aggregation network, is employed within the YOLOv7 architecture to optimize memory access costs and GPU computing efficiency. In order to prevent information over-inflation and loss during multi-layer propagation, the ELAN network facilitates interaction between layers, enabling deeper network depth and improved accuracy. The ELAN network structure controls connection paths of varying lengths to promote effective network learning and convergence. YOLOv7 incorporates module-level reparameterization techniques that break modules into several equivalent microstructures with consistent parameter transformations, which greatly enriches training resources and enhances model performance while maintaining prediction consistency with previous YOLO iterations. The YOLOv7 architecture retains the three-part structure of its predecessors—including the backbone, neck, and YOLOHead—and is illustrated in Figure 3.

The backbone network serves as the primary feature extractor in YOLOv7, extracting image inputs and generating corresponding feature layers. Three effective feature layers are retained in the backbone section for use in subsequent network construction. These layers represent the feature sets of input images and serve as a critical element to inform network performance.

The neck network, which acts as an enhanced feature extractor, merges the three effective feature layers generated in the backbone section in order to amalgamate feature information from various scales. The Panet structure remains employed in YOLOv7, enabling both up-sampled and down-sampled feature fusion. In the neck section, feature extraction continues through utilization of the effective layers obtained previously.

Finally, the YOLOHead function serves as both classifier and regressor, enabling access to the three enhanced effective feature layers generated by the backbone and neck. Specifically, YOLOHead assesses whether a priori boxes correspond to objects detected in feature points. As with earlier iterations of YOLO, the decoupling head remains integrated in YOLOv7, executing classification and regression through a 1 × 1 convolution approach.

Despite the performance improvements in YOLOv7 compared to its predecessors, it still faces challenges in detecting small targets and performing target detection tasks in complex backgrounds. Consequently, it fails to meet the specific requirements of this study, which focuses on detecting air conditioner external units in street view image data. Therefore, targeted improvements are necessary.

## 3. Results

### 3.1. SC4-YOLOv7 Algorithm Improvement

The YOLOv7 algorithm is a new algorithm of the YOLO series proposed in 2022 and is a one-stage detector with very good performance, so it is chosen as our base model. YOLOv7 requires pre-training weights on large-scale data sets and is less effective in recognizing air conditioner external units in complex backgrounds in street view image data. Therefore, in this study, we optimize and improve the initial YOLOv7 model in three aspects, namely, the backbone network, enhanced feature extraction network, and detection head, to form a new algorithm for efficient extraction of air conditioner external units in complex background environment of street view image data, in response to the small size of air conditioner external unit targets in street view images and the fact that they are easily obscured and other features are not obvious.

#### 3.1.1. Backbone Network Optimization—Introducing SimAM Parameter-Free Attention

The visual attention mechanism, a unique signal processing mechanism in the human brain, is an inherent component of human vision. It enables rapid scanning of the global image to identify the area requiring focus, commonly referred to as the attention focus. Subsequently, it allocates additional attention resources to this area, enabling the capture of more detailed information while suppressing peripheral, irrelevant details. Given the complex characteristics of street view image data, this algorithm incorporates an attention mechanism module to mitigate the impact of complex backgrounds on detection results. This approach helps the model prioritize the extraction of air conditioner external unit features and enhances learning regarding these features within relevant image regions.

While some existing attention modules refine features solely in either the spatial or channel dimensions, their limited flexibility becomes evident when both undergo simultaneous change. The SimAM module addresses this challenge by serving as a parameter-free 3D attention module that does not require additional parameters to be added to the original network. In contrast to one-dimensional (1D) channel attention and two-dimensional (2D) spatial attention modules, which focus only on channel or spatial location importance, respectively, 3D attention enables simultaneous channel and spatial location feature attention. This approach infers 3D attention weights by analyzing the feature mapping, allowing for consideration of both channel and spatial location importance simultaneously.

SimAM 3D weights implement a visual neurological theory that acknowledges neurons’ unique firing patterns and spatial depression effects in visual processing tasks. Neurons demonstrating spatial depression effects should be assigned higher weights, and the easiest means of identifying these neurons is measuring linear differentiability between the target neuron and its surrounding ones. Thus, an energy function is defined for each neuron based on said scientific findings. If an input image *R* with input features *X*∈*R* *C* × *H* × *W* exists in *C* channels and *M* = *H* × *W* neurons, each channel can theoretically have *M* energy functions. However, to reduce iterative computation and avoid the need to calculate the mean and variance for each position, the average mean *μ* and variance *σ* of all neurons *x_i_* from a single channel can be calculated using Equations (4) and (5).

(4) $$\hat{\mu }=\frac{1}{M}\sum_{i=1}^{M}x_{i}$$

(5) $${\hat{\sigma }}^{2}=\frac{1}{M}\sum_{i=1}^{M}{\left(x_{i}-\hat{\mu }\right)}^{2}$$

Therefore, the final minimum energy function is shown in Equation (6):

(6) $$e_{t}^{*}=\frac{4\left({\hat{\sigma }}^{2}+\lambda \right)}{{\left(t-\hat{\mu }\right)}^{2}+2{\hat{\sigma }}^{2}+2\lambda }$$

where *t* is the target neuron and *λ* is the hyperparameter.

From Equation (3), SimAM can be evaluated for each neuron of each network by defining the energy function of linear differentiability and calculating the minimum energy $e_{t}^{*}$. The lower the energy of $e_{t}^{*}$, the more the neuron *t* is distinguished from the surrounding neurons and the higher the importance. Therefore, the importance of neurons can be obtained by $1/e_{t}^{*}$. Finally, based on the gain effect of neuron response, the weighting is performed by using the deflation operation, while the sigmoid function can also limit the excessive values of *E* (features grouped by neuron importance $1/e_{t}^{*}$) but also does not affect the relative importance of each neuron, as in Equation (7).

(7) $$\tilde{X}=sigmoid\left(\frac{1}{E}\right)⊙X$$

When recognizing image features, both channel and spatial location are essential, and therefore, this paper introduces parameter-free attention SimAM to the ELAN of the backbone network to evaluate feature importance comprehensively and effectively. By leveraging the energy function, features extracted from the backbone can be evaluated, and important neurons carrying rich messages can be identified. This improved model enables the identification of crucial features, the suppression of irrelevant feature interference, improvement in the network’s feature representation capability, and enhancement of the model’s target localization ability to focus on feature representation that facilitates recognition of air conditioner external unit targets in street view image data.

#### 3.1.2. Neck Network Optimization—Introducing Coordinate Attention

The Coordinate Attention (CA) mechanism efficiently embeds location information into channel attention, allowing the CA module to pay attention to both regions of interest in the channel and their corresponding locations. This two-step approach involves information embedding and attention weight generation.

Traditional attention mechanisms, including SENet [36], ECA [37], CBAM [38], etc., usually use global pooling to globally encode spatial information, which ignores location information. In order to accurately capture both channel information and location information, the information embedding stage first uses pooling kernels of size (*H*, 1) and (1, *W*) for each channel in the horizontal and vertical directions, respectively, for the feature layers input to the CA module. Better capture of location information and channel information with a small increase in computational effort facilitates the network to locate the target of interest. The pooling outputs for the cth channel with height *H* and the *c*th channel with width *W* are as follows:

(8) $$Z_{C}^{h}\left(h\right)=\frac{1}{W}\sum_{0\leq i\leq W}x_{c}\left(h,i\right)$$

(9) $$Z_{C}^{w}\left(w\right)=\frac{1}{H}\sum_{0\leq j\leq H}x_{c}\left(j,w\right)$$

The features in the horizontal and vertical directions of the feature map are represented by $x_{c}\left(h,i\right)$ and $x_{c}\left(j,w\right)$, respectively. The horizontal and vertical tensors, $Z_{C}^{h}\left(h\right)$ and $Z_{C}^{w}\left(w\right)$, respectively, are obtained after pooling. These tensors are then stitched together and transformed using a 1 × 1 convolution function, *F*_1_, as follows:

(10) $$f=\delta \left(F_{1}\left(\left[Z^{h},Z^{w}\right]\right)\right)$$

In the given equation, *δ* is the nonlinear activation function, […, …] denotes the splicing operation, and *f* is the intermediate feature for encoding spatial information in the horizontal and vertical directions. The f is decomposed into two separate tensors $f_{h}$ and $f_{w}$ in horizontal and vertical directions, and finally the number of channels of the two tensor is made consistent by a 1 × 1 convolution *F*. Using the sigmoid activation function *σ* outputs,

(11) $$g^{h}=\delta \left(F\left(f_{h}\right)\right)$$

(12) $$g^{w}=\delta \left(F\left(f_{w}\right)\right)$$

The output results $g^{h}$ and $g^{w}$ of the above equation are the weights of attention in the vertical and vertical directions, respectively. The initial input feature $x\left(i,j\right)$ is multiplied with the weights of the corresponding positions to obtain the output result $y\left(i,j\right)$, and the final output of the CA module is as follows:

(13) $$y\left(i,j\right)=x\left(i,j\right)*g^{h}\left(i\right)*g^{w}\left(j\right)$$

The objective of our study is to enhance the YOLOv7 network’s ability to capture target features in street view image data of air conditioner external units located outside. To achieve this goal, a Coordinate Attention module is incorporated into the network’s neck. The inclusion of this module ensures that the network focuses on the location and channel information of the relevant target features, thus enabling better feature extraction.

#### 3.1.3. Head Network Optimization—Add Small Target Detection Layer Head

Compared with conventional targets, small targets are characterized by weak features and little information, and it is very difficult to distinguish them from similar backgrounds or contiguous targets. When facing the problems of complex environments such as low illumination and shadow occlusion, the task of detecting small targets in street view image data poses a higher challenge.

The original YOLOv7 model backbone network was downsampled a total of five times to obtain five layers of feature expressions (P1, P2, P3, P4, and P5), where Pi denotes a resolution of 1/2i of the original image, and although multi-scale feature fusion was achieved in the neck network through top-down and bottom-up aggregation paths, it did not affect the scale of the feature maps, and the final detection head was partially in the detection head induced through P3. The detection of the target is performed on the detection heads led through the three-level feature maps P3, P4, and P5, whose feature map scales are 80 × 80, 40 × 40, and 20 × 20, respectively. For the convenience of expression, the detection heads led through the Pi-level feature maps are referred to as Pi-level detection heads hereafter.

The self-built street view image data discussed in this paper contain more distant individual targets of air conditioner external units, whose scales are often smaller than 20 × 20 pixels. These targets lose most of their feature information after repeated downsampling, and thus it remains challenging to detect them even by the higher resolution P3 layer detection head. To address this issue and achieve better detection results for these tiny targets, we introduce a new detection head on the YOLOv7 model that utilizes the P2 layer features. The resolution of the P2 layer detection head is 160 × 160 pixels, equivalent to only two downsampling operations in the backbone network, containing richer underlying feature information of the targets. The two P2 layer features obtained from top-down and bottom-up paths in the neck network are fused with the same scale features in the backbone network through concatenation. The output features are the fusion results of the three input features, making the P2 layer detection head fast and effective in dealing with tiny targets. Moreover, the newly added detection head is specific to the underlying features and generated from a low-level, high-resolution feature map that is more sensitive to tiny targets. Although the addition of this detection head increases the model computation and memory usage, it effectively mitigates the negative impact of scale variance. The P2 detection head, together with the original three detection heads, can effectively improve the detection capability for tiny targets.

Through practical tests, the actual results also prove that after adding the small target detection layer, the model detects a significant increase in the individual detection rate of small targets in street view image data.

#### 3.1.4. Building the New SC4-YOLOv7 Algorithm

**Figure 4:**
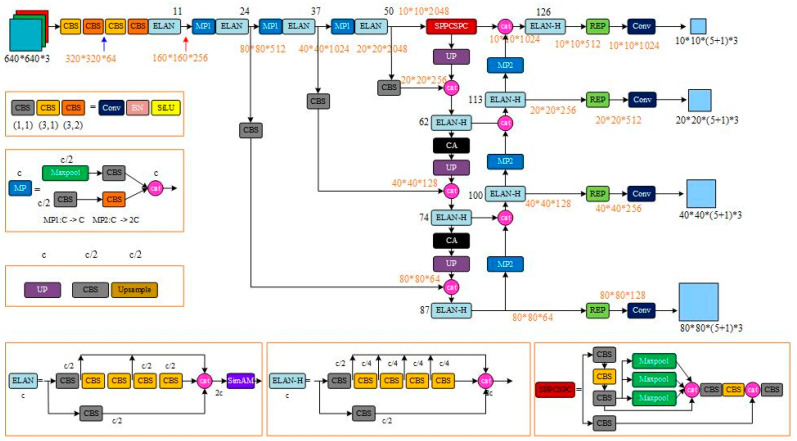
SC4-YOLOv7 network structure diagram.

In this paper, we propose a new SC4-YOLOv7 model that is optimized and improved in three parts: the original YOLOv7 model backbone network, enhanced feature extraction network, and detection head. The purpose of these changes is to make the model more effective in detecting targets in complex background environments and tiny targets in street view image data. Figure 4 shows the structure of the network after these modifications.

The SC4-YOLOv7 model comprises four parts: input, backbone network, neck network, and detection head. The input part enhances the training data with techniques like Mosaic and applies adaptive scaling and anchor frame calculation to images. To address issues such as similar backgrounds in street view images, difficult feature extraction due to texture replication, and loss of network propagation feature information, we introduced the SimAM attention module to the backbone network of the original YOLOv7 model. This module utilizes an energy function to identify effective features and suppress irrelevant ones, thereby improving the feature extraction capability of the backbone network for small targets. The neck network is based on the SPP + PAN structure but includes a coordinate attention mechanism for feature fusion to obtain hierarchical feature representations that are passed on to the detection head. A small target detection layer is added to the detection head to provide four detection scales (10 × 10, 20 × 20, 40 × 40, and 80 × 80). Finally, the optimal prediction frame is determined by non-maximal suppression (NMS) of the target detection frame.

### 3.2. Research on Extraction of Air Conditioner External Units from Street View Images

#### 3.2.1. Experimental Environment Configuration

In this study, all comparison models perform detection operations on the GPU server. Table 1 shows the experimental configuration. A total of 5680 images of individual air conditioner external units were used for training during the detection process. The stochastic gradient descent (SGD) momentum of all target detection algorithms in this experiment was set to 0.9. The initial learning rate is set to 0.01, the weight decay is 0.0005, and the training is performed using a pre-training strategy. The input size is fixed to the same size as used in the detection process (Table 2).

#### 3.2.2. Evaluation of the Performance of Air Conditioner External Unit Extraction of Street View Image Data

**Figure 5:**
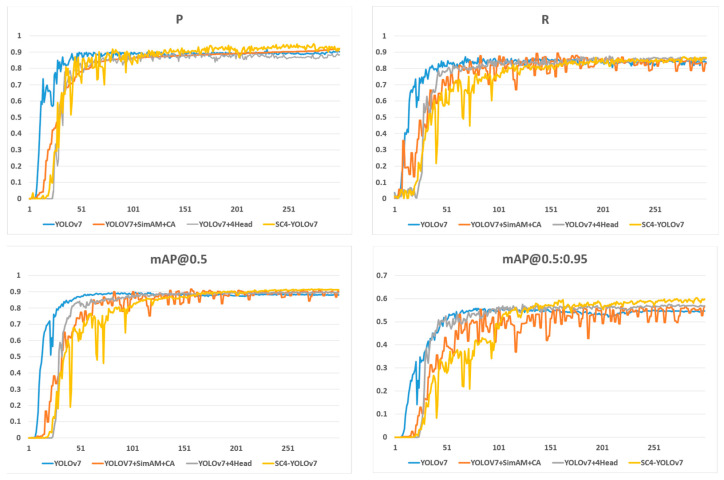
Each algorithm detects the experimental effect.

**Figure 6:**
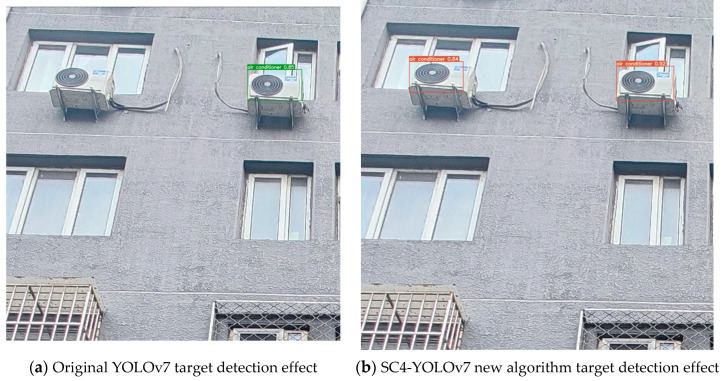
Comparison chart of the effect of the target detection task of air conditioner external unit.

The experimental comparison indicates that the small target detection layer improves the recall from 0.8397 to 0.8588, mAP@0.5 from 0.8793 to 0.8945, and mAP@0.5:0.95 from 0.5466 to 0.5681 in the original YOLOv7 model despite a slight reduction in precision. With the addition of SimAM and CA attention mechanisms, the model’s comprehensive detection ability improves with little effect on recall rate but with increases in precision, mAP@0.5, and mAP@0.5:0.95 indexes from 0.9024, 0.8793, and 0.5466 to 0.9198, 0.9017, and 0.5683, respectively. By combining the small target detection layer with SimAM and CA attention mechanisms, the model exhibits further improvement in detection ability, with precision, recall, mAP@0.5, and mAP@0.5:0.95 indexes reaching 0.922, 0.8660, 0.9121, and 0.5977 (Table 3). The model performs well in detecting air conditioner external units, even under challenging conditions such as large target individuals at close distances, small target individuals at long distances, and individuals blocked by trees and fences. Compared with the original YOLOv7 algorithm, the new SC4-YOLOv7 algorithm shows significant improvements in detecting target objects and can detect many objects that were previously missed. Figure 5 provides a comparison of the training process. Figure 6 provides a comparison of the results.

**Figure 7:**
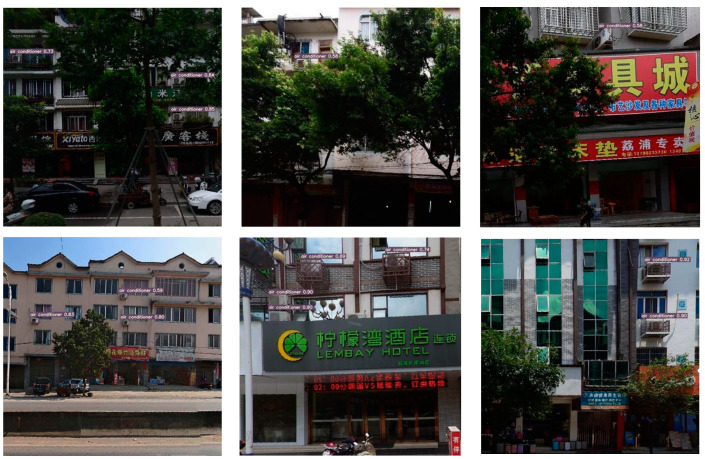
The effect of detecting the air conditioner external unit target under different conditions such as obscured by fence, obscured by tree, oblique direction position, and small target from a distance in street view image data.

The SC4-YOLOv7 network model was used to detect the air conditioner external units on all the acquired street view image data of the main roads in Guilin, Guangxi Zhuang Autonomous Region, and the results achieved the expected goal: the air conditioner external units were accurately detected. Some of the results are shown in Figure 7.

### 3.3. Spatial Distribution of Urban Air Conditioner External Units

**Figure 8:**
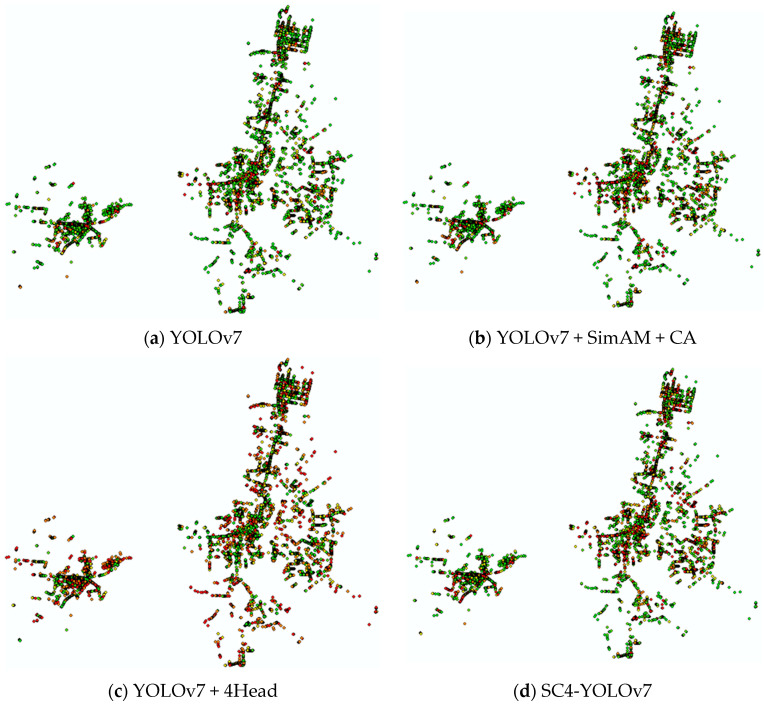

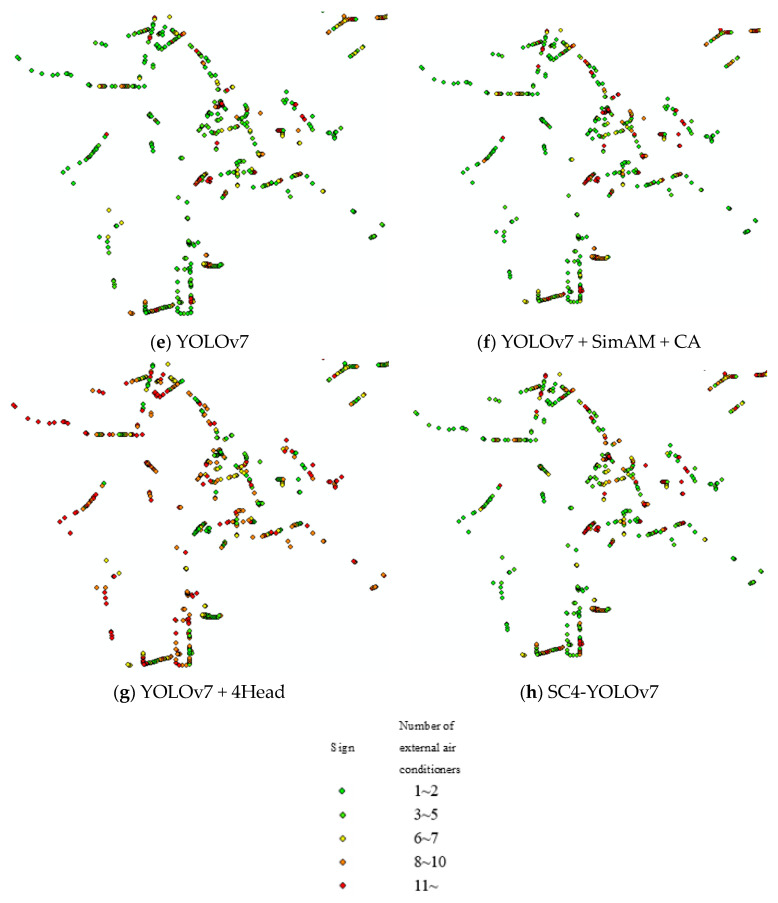
Air conditioner external space distribution map.

The new data combination is formed by counting the number of air conditioner external units detected in each street view image and combining the corresponding coordinate information. Using ArcGIS, an air conditioning statistical map is created based on geographical location and the number of detected frames, which corresponds to the number of detected air conditioner external units. In Guilin city, there are 61,929 coordinate points of road networks and 123,858 street view image data. The use of the YOLOv7 algorithm results in the detection of 13,806 street view image data, 11,752 coordinate points, and 87,176 air conditioner external units, while the YOLOv7 + SimAM + CA algorithm detects 13,873 street view image data, 11,805 coordinate points, and 99,234 air conditioner external units. Furthermore, using the YOLOv7 + 4Head algorithm, 13,959 street view images, 11,869 coordinate points, and 114,118 air conditioner external units are detected, while the SC4-YOLOv7 algorithm detects 13,933 street view images, 11,850 coordinate points, and 113,553 air conditioner external units. Notably, the SC4-YOLOv7 algorithm exhibits superior performance in detecting 113,553 air conditioner external units in 13,933 street view images with 11,850 coordinate points. Based on the detection statistics of each algorithm, a distribution map of air conditioners is generated, and part of the situation is shown in Figure 8.

As can be clearly seen from the detection results point diagram, when the original YOLOv7 algorithm joins the small target detection layer, the network can pay more attention to the tiny target individuals and the checking of all rates by the algorithm increases significantly, so the image indicates a significantly greater number of orange and red dots. However, relatively, the algorithm accuracy will have a certain degree of reduction, that is, a large number of small target individuals that are not air conditioner external units will be used in air conditioner external unit detection. The YOLOv7 network with the attention mechanism can better focus on the required air conditioner external unit features from the complex background environment of the street view images, correctly detect and identify them, and improve the accuracy of the algorithm. The SC4-YOLOv7 algorithm in this study combines the above advantages and is generally optimal considering the accuracy of the detection under the condition of ensuring few or even no omissions.

The images obtained by combining the coordinate data of the main road network of Guilin city and the statistical data of the number of target detection of air conditioner external units in street view images can reflect the high coverage and concentrated distribution of local air conditioners very intuitively and provide corresponding data support for the policy formulation and method implementation of heat wave disaster prevention.

## 4. Discussion

Street view image data provide researchers with a low-cost, efficient, and wide-coverage way to record and observe the physical urban environment from a parallel human perspective [39]. In combination with the rapidly developing field of artificial intelligence, the ability to collect, process, compute, and analyze large-scale data in a short period has increased dramatically and has now become an important data source for urban application research, bringing new methods and ideas [40].

The assessment and analysis of air conditioner coverage and utilization, serving as crucial indicators in the context of addressing urban heat wave disasters, primarily rely on outdated manual techniques. Street view image data, which offer a vibrant reflection of a city’s status, have been overlooked as a potential source of information. Currently, available artificial intelligence algorithms, catering to a broad spectrum of applications, have not been specifically implemented for the targeted recognition of air conditioner external units in street view image data. Consequently, this study presents a deep-learning-oriented SC4-YOLOv7 algorithm for the precise detection and counting of air conditioner external unit targets on buildings within street view image data. This approach offers a more convenient and efficient solution for related research endeavors.

Although this study only focuses on the target detection and counting statistics of air conditioner external units in urban street view image data, the overall method can be fully migrated to the detection of other target objects in street view image data with complex background environment conditions that are also obscured by trees and fences and small targets of different sizes and distances that are difficult to detect, and the idea of applying street view image data efficiently and combining them with location coordinate information will support future multi-scale and multi-dimensional urban information mining and extraction, and urban residential environment enhancement.

As street view image data are gathered by the street view collection vehicles, equipped with panoramic equipment, while traversing city roads, the current coverage of both Google Street View and Baidu Panorama services remains limited. At this stage, the street view image data primarily cover major city roads. However, data from smaller roads and residential communities, where vehicles face difficulty navigating, are essentially unavailable, which has also led to the inability to complete detail-demanding and relevant urban studies based on Street View image data alone. Our study’s street view image data are based solely on the information of the city’s main road network in Guilin City, which unfortunately cannot comprehensively represent the entire urban environment. Nonetheless, the counting results from our research provide a sufficient macroscopic perspective to reveal the proportional density of air conditioning coverage and its spatial distribution across the city. Moving forward, we will continue to refine and expand the data scope by covering areas that are currently excluded, such as selecting residential communities and deploying professionals to take additional photographs of air conditioner external units located on interior building walls, among other improvements.

However, street view image data are inherently limited as they can only capture the physical environment that is visible to the human eye in urban areas. These data struggle to effectively represent natural environment characteristics such as urban air, soil, and water conditions. Therefore, future studies focusing on urban applications based on street view images will undoubtedly require the integration of multiple data sources, including remote sensing images, geotagged social media data, and more. Remote sensing images, obtained through remote sensing technology, offer an overhead view of the ground [41,42,43], enable the visualization of landform shapes, and possess the advantages of providing a macroscopic, timely, and rapid acquisition of features. However, current urban applications that combine street view images and remote sensing data largely focus on urban greening, a domain that is nearing saturation. There is a significant lack of research addressing other natural environments. Geotagged social media data reflect the environmental situation from both rational and emotional perspectives [44,45,46], and when combined with street view image data, offer a more comprehensive analysis of target areas. However, the dispersed and voluminous nature of social media data with geographical locations presents challenges in data extraction and filtering, limiting the number of studies that incorporate these data.

In our future work, we plan to utilize data sources that are not limited to web-based street view images. Specifically, we aim to integrate multiple data sources to expand our analysis capabilities, such as incorporating subjective social media photos with geographic location data in conjunction with street view images. Additionally, we will leverage data sources containing more subjective emotions, such as questionnaires, to better understand the direct impact of environmental factors on residents. Remote sensing image data serve as a complementary source to street view image data, offering higher-level and more macroscopic information. We propose combining remote sensing data with natural data sources [47], including geothermal and air environment data, as well as humanistic data sources, such as AOI, rooftop, and pedestrian flow data, to enhance urban multi-source data mining, information extraction, and provide powerful support for urban disaster prevention and mitigation, environmental analysis, rational planning, and intelligent construction.

## 5. Conclusions

In this study, we utilized continuous street view image data collected from both sides of the city’s main roads to detect air conditioner external units on buildings. We identified the limitations of the original YOLOv7 algorithm model in extracting information about these external units and addressed them. To improve the detection accuracy of air conditioner external unit recognition for street view image data under various complex environmental conditions, we developed a new algorithm called SC4-YOLOv7. This algorithm incorporates several enhancements, including the addition of SimAM and CA attention mechanisms, as well as the inclusion of small target detection layers.

Compared to the original YOLOv7 algorithm, the proposed SC4-YOLOv7 algorithm demonstrates significant improvements in detecting air conditioner external unit targets in street view image data. Specifically, it improves the detection precision rate from 90.24% to 92.2%, the detection recall rate from 83.96% to 86.6%, and the mean average precision from 87.93% to 91.21%. Therefore, the improved SC4-YOLOv7 model not only ensures accurate detection but also enables efficient statistical analysis. It can serve as a valuable tool in future applications, such as detecting air conditioner external unit information in large-scale urban street view images and effectively responding to high-temperature and heat wave disasters.

## Figures and Tables

**Table 1:** Comparison of evaluation indexes of detection effect of basic algorithms.

Algorithm	P	R	mAP@0.5	mAP@0.5:0.95
YOLOv5	0.8760	0.7389	0.8441	0.5080
YOLOv7	0.9024	0.8397	0.8793	0.5466
YOLOv8	0.9053	0.8434	0.8829	0.5487

**Table 2:** Experimental configuration.

Configuration	Parameter
System Environment	ubuntu20.04
GPU	V100-SXM2-32GB
Accelerated environment	CUDA 11.3
Libraries	PyTorch1.11.0
Language	Python 3.8

**Table 3:** Comparison of evaluation indexes of detection effect of each model.

Algorithm	P	R	mAP@0.5	mAP@0.5:0.95
YOLOv7	0.9024	0.8397	0.8793	0.5466
YOLOv7 + SimAM + CA	0.9198	0.8317	0.9017	0.5683
YOLOv7 + 4Head	0.8822	0.8588	0.8945	0.5681
SC4-YOLOv7	0.9220	0.8660	0.9121	0.5977

## Data Availability

The data that support the findings of this study are not openly available due to reasons of sensitivity and are available from the corresponding author upon reasonable request.
